# Neutrophil-to-lymphocyte ratio predicts early cognitive decline associated with tau pathology in Alzheimer’s disease: a longitudinal study of the ADNI cohort

**DOI:** 10.3389/fnagi.2026.1872617

**Published:** 2026-09-16

**Authors:** Xin Wang, Xiaolei Liu, Zhigang Wang, Chenle Jia

**Affiliations:** 1 Department of Neurology, Third Hospital of Shanxi Medical University, Shanxi Bethune Hospital, Shanxi Academy of Medical Sciences, Tongji Shanxi Hospital, Taiyuan, China; 2 Clinical Research Center for Neurological Diseases of Guangxi Province, Guilin Medical University, Guilin, China; 3 Key Laboratory of Environmental Exposomics and Entire Lifecycle Health of Guangxi Province, Guilin Medical University, Guilin, China; 4 Key Laboratory of Brain and Cognition of Guangxi Province, Guilin Medical University, Guilin, China; 5 Key Laboratory of Diabetic Systems Medicine of Guangxi Province, Guilin Medical University, Guilin, China; 6 Head and Neck Surgery Department, Shanxi Province Cancer Hospital/Shanxi Hospital Affiliated to Cancer Hospital, Chinese Academy of Medical Sciences/Cancer Hospital Affiliated to Shanxi Medical University, Taiyuan, China

**Keywords:** Alzheimer’s disease, cognitive decline, mild cognitive impairment, neutrophil-to-lymphocyte ratio, tau pathology

## Abstract

**Background:**

Neuroinflammation is a key driver of Alzheimer’s disease (AD). Although the neutrophil-to-lymphocyte ratio (NLR) is an easily accessible peripheral inflammatory marker, yet its longitudinal association with domain-specific cognitive decline and the underlying neuropathological mechanisms during the preclinical and early stages of AD remain unclear.

**Methods:**

A total of 1,190 non-people living with dementia older adults from the AD Neuroimaging Initiative cohort were included, comprising 405 cognitively normal participants and 785 participants with mild cognitive impairment (MCI). Participants were stratified according to baseline NLR (≥ 3versus < 3). Linear mixed-effects models were used to examined the association between baseline NLR and longitudinal decline across multiple cognitive domains. Cox proportional hazards model assessed progression risk to MCI or AD. Mediation analyses evaluated whether cerebrospinal fluid (CSF) biomarkers, including amyloid-β_42_ (Aβ_42_), total tau (t-tau), and phosphorylated tau (p-tau), statistically accounted for the association between NLR and cognitive decline.

**Results:**

During follow-up, a high baseline NLR (≥ 3) was independently associated with accelerated decline in global cognition, memory, executive function, language, and visuospatial abilities (all *p* < 0.05), as well as a 20% higher risk of clinical progression per 1-unit increase in NLR (hazard ratio = 1.20, 95% confidence interval: 1.12–1.29, *p* = 0.001). Mediation analyses indicated that CSF t-tau and p-tau, but not Aβ_42_, partially explained the association between NLR and cognitive decline. The observed associations remained robust across sensitivity and subgroup analyses.

**Conclusion:**

Elevated NLR was significantly and independently associated with accelerated decline across multiple cognitive domains and an increased risk of clinical progression in older adults at risk for AD. The observed associations with tau pathology are consistent with a potential role for peripheral immune dysregulation in early disease trajectories; however, formal predictive modeling and external validation are required before clinical application.

## 1 Introduction

Global population aging represents one of the most profound societal shifts of the 21st century, with the number of individuals aged 60 years and older projected to reach 2.1 billion by 2050. This demographic transition is particularly pronounced in developing countries and is accompanied a rapid increase in the prevalence of age-related neurodegenerative disorders, particularly dementia (Moeteke, 2022; Yang et al., 2024). Alzheimer’s disease (AD), the leading cause of dementia, is strongly associated with aging. Globally, more than 55 million people are currently living with dementia, and this number is projected to increase to 152 million by 2050 (Meyers et al., 2023). China bears a particularly substantial burden, with the prevalence of dementia projected to increase 2.13-fold by 2050, reaching 35.98 million compared with 2015 estimates (Wang et al., 2019). Beyond its profound impact on individual health, AD imposes a substantial socioeconomic burden. Accordingly, identifying actionable and modifiable risk factors to prevent or delay disease progression remains a pressing public health priority.

Immune system dysregulation plays a key role in promoting AD pathogenesis and has recently attracted considerable research interest. AD is increasingly recognized as a systemic disorder characterized by dynamic crosstalk between peripheral and central immune responses (Liu et al., 2023; Bettcher et al., 2021; Zhang et al., 2025). Accumulating evidence indicates that peripheral immune dysregulation may exacerbate neuroinflammation, thereby promoting AD pathology (Bayraktaroglu et al., 2025; Walker et al., 2023; Cao et al., 2025). Specifically, aberrant peripheral immune cells can infiltrate the central nervous system (CNS) through a compromised blood–brain barrier (BBB), potentially intensifying neuroinflammation and accelerating disease progression (Zhang et al., 2025; Zhang et al., 2024). Furthermore, elevated lymphocyte levels have been observed identified at sites of amyloid-β (Aβ) plaques and tau aggregates in the brain. In contrast, phenotypic alterations in lymphocytes have been reported in cerebrospinal fluid (CSF) and peripheral blood, underscoring the contribution of adaptive immunity to disease progression (Jacobs et al., 2024). Moreover, peripheral cytokine profiles are altered in patients with AD, characterized by markedly increased concentrations of pro-inflammatory cytokines, including interleukin (IL)-1β, IL-6, and tumor necrosis factor-alpha (Hesse et al., 2016), together with reduced concentrations of anti-inflammatory cytokines, such as IL-10 and IL-13 (Mohd Hasni et al., 2016). Notably, Graff et.al demonstrated that immune dysregulation may occur before the development of the canonical pathological hallmarks of AD, including Aβ-associated senile plaques and tau-based neurofibrillary tangles (Graff-Radford et al., 2021).

The neutrophil-to-lymphocyte ratio (NLR), a simple and cost-effective biomarker derived from peripheral blood counts, reflects the systemic balance between innate and adaptive immunity. Despite its potential utility, studies investigating the association between NLR and AD or mild cognitive impairment (MCI) have yielded inconsistent findings. Although several studies have associated elevated NLR with AD or MCI severity, CSF amyloid and tau biomarkers, and neuroinflammation (Jacobs et al., 2024; Evlice et al., 2023; Algul and Kaplan, 2024; Giannelli et al., 2023; Aprile et al., 2025), others have reported that these associations are no longer significant after adjustment for apolipoprotein E *(APOE)* ε4 genotype and sociodemographic factors (Hong et al., 2022; Kara et al., 2021; Rembach et al., 2014). A major limitation of the current evidence is that most studies have focused on individuals with end-stage disease, potentially confounding the effects of aging and chronic comorbidities, including obesity, diabetes, and hypertension, all of which are independently associated with elevated NLR and AD progression. Moreover, few studies have investigated the association between NLR and decline across specific cognitive domains. Consequently, the biological mechanisms linking peripheral inflammation to AD pathogenesis remain incompletely understood. In particular, whether the core pathological features of AD, namely Aβ and tau, mediate the association between NLR and cognitive risk remains unclear.

Therefore, the present study sought to address these knowledge gaps in a well-characterized cohort of non-people living with dementia individuals comprising those with MCI and cognitively normal (CN) participants. Specifically, this study aimed to (1) investigate the cross-sectional and longitudinal association between baseline NLR and cognitive decline across multiple domains; (2) determine whether baseline NLR is associated with the risk of incident MCI or AD; (3) examine whether CSF AD biomarkers, including Aβ_42_, total tau (t-tau), and phosphorylated tau (p-tau), statistically account for a proportion of the association between NLR and cognitive decline; (4) evaluate the consistency of these associations across key demographic and clinical subgroups.

## 2 Methods

### 2.1 Participants

Data were obtained from the AD Neuroimaging Initiative (ADNI) database (ClinicalTrials.gov identifier: NCT00106899; accessed in November 2025; adni.loni.usc.edu), comprising participants from ADNI GO, ADNI-1, ADNI-2, and ADNI-3 cohorts. ADNI is a multicenter, longitudinal observational study initiated in 2003 to evaluate whether neuroimaging, neuropsychological assessments, genetic factors, clinical measures, and biochemical biomarkers can be integrated to monitor the progression of MCI and early AD. The ADNI study was conducted in accordance with the Declaration of Helsinki, and the study protocol was approved by the institutional review boards at each participating center. Participants were older adults aged 55–90 years and were classified as CN, having MCI, or having AD. The inclusion criteria were as follows: (1) ADNI participants were classified as CN or had MCI at study entry; (2) participants completed at least two follow-up visits; (3) participants had available baseline NLR measurements together with neuropsychological assessments data collected at baseline and during annual follow-up. Participants with major psychiatric or neurological disorders were excluded. Consequently, the primary analysis included 405 CN participants and 785 participants with MCI. Written informed consent was obtained from all participants or their authorized representatives at each participating site. Detailed information regarding the ADNI study, including the inclusion and exclusion criteria, is available at https://adni.loni.usc.edu/.

### 2.2 Calculation of NLR from complete blood count

Following an overnight fast, venous blood samples were collected in the morning by trained personnel using ethylenediaminetetraacetic acid anticoagulant tubes. The samples were processed on the day of collection to obtain a complete blood count, including absolute neutrophil and lymphocyte counts. NLR was calculated as the absolute neutrophil count divided by the absolute lymphocyte count.

### 2.3 Measurement of CSF-based biomarkers of AD

The CSF samples were collected from 853 participants by lumbar puncture following a minimum 6-h fast. The samples were subsequently transported to the ADNI Biomarker Core Laboratory at the University of Pennsylvania Medical Center for analysis. Concentrations of the core AD biomarkers—including Aβ_42_, p-tau, and t-tau—were quantified using the Roche Elecsys immunoassay on the cobas e 601 automated analyzer systems (F. Hoffmann-La Roche Ltd., Basel, Switzerland) according to established protocols. Detailed procedures for CSF collection, handling, and biomarker analysis are described in ADNI procedures manuals for the respective study phases (available at: http://www.adni-info.org).

### 2.4 Cognitive assessments

Cognitive function was assessed at baseline and all follow-up visits using established neuropsychological instruments. Global cognition was evaluated using the Mini-Mental State Examination (MMSE), with higher scores indicating better cognitive performance. Disease severity was assessed using the Clinical Dementia Rating Scale Sum of Boxes (CDR-SB), with higher scores indicating greater cognitive impairment. Cognitive performance was further evaluated using the AD Assessment Scale-Cognitive Subscale (ADAS-Cog), administered in its 11-item (ADAS11) and 13-item (ADAS13) versions, with higher scores reflecting poorer cognitive function. Memory was assessed using the ADAS Delayed Word Recall subscale (ADAS-Q4). Episodic memory was evaluated using the Rey Auditory Verbal Learning Test (RAVLT), which assesses immediate recall, learning across trials, and percentage of forgetting. Higher scores for immediate recall and learning across trials indicate better performance, whereas higher percentage forgetting scores indicate poorer memory performance. Functional abilities in daily living were assessed using the Functional Assessment Questionnaire (FAQ), with higher scores indicating greater functional impairment.

Standardized scores for the ADNI-modified Preclinical Alzheimer’s Cognitive Composite (PACC), incorporating the Digit Symbol Substitution Test (mPACCdigit) and Trail Making Test Part B, were also evaluated with higher scores indicating better cognitive performance. Additionally, four ADNI domain-specific composite scores were employed: ADNI memory (ADNI-MEM), ADNI executive function (ADNI-EF), ADNI language (ADNI-LAN), and ADNI visuospatial function (ADNI-VIS). These composite variables were derived from latent variable modeling of multiple cognitive tests to provide robust summary measures of specific cognitive domains.

### 2.5 Diagnostic criteria and longitudinal cognitive status definition

The CN participants were required to have an MMSE score of 24–30, a global CDR score of 0, no subjective memory concerns, and objective memory performance above the education-adjusted cutoffs on the Logical Memory II subscale. Participants with MCI were required to have an MMSE score of 24–30, a global CDR score of 0.5 with a memory box score of ≥ 0.5, objective memory loss on the Logical Memory II subscale, and largely preserved activities of daily living. Participants with AD met the National Institute of Neurological and Communicative Disorders and Stroke and the AD and Related Disorders Association criteria for probable AD (McKhann et al., 1984), with an MMSE score of 20–26, a global CDR score of ≥0.5, subjective memory concerns, functional impairment, and abnormal memory performance according to education-adjusted cutoffs. Based on serial clinical diagnoses, participants were classified as cognitively stable or cognitively progressive. The cognitively stable group comprised participants with stable CN, stable MCI, or reversion from MCI to CN. In contrast, the cognitively progressive group included participants who progressed from MCI to dementia or from CN to MCI or dementia.

### 2.6 *APOE* genotyping

Genomic DNA was extracted from peripheral blood samples using the QIAamp^®^ DNA Blood Mini Kit, followed by polymerase chain reaction amplification. *APOE* genotypes were determined by allelic discrimination assays targeting the two *APOE* single-nucleotide polymorphisms (rs7412 and rs429358) using restriction fragment length polymorphism technology.

### 2.7 Statistical analyses

Participants were stratified into two group according to a predefined NLR cutoff of 3: The Normal NLR group (NLR < 3) and the High NLR group (NLR ≥ 3). This threshold has been widely adopted in clinical and epidemiological studies as an indicator of systemic inflammatory burden and has also been applied in previous AD research (Wang et al., 2025; Abete et al., 2019; Yap et al., 2018; Cespedes Feliciano et al., 2017). Within each NLR subgroup, participants were further classified as cognitively progressive or cognitively stable based on their longitudinal clinical diagnoses. To evaluate the robustness of this classification, supplementary sensitivity analyses were performed using alternative NLR cutoffs of 2.5 and 3.5. Baseline demographic and clinical characteristics were compared between groups using independent *t*-tests, chi-square tests, or non-parametric tests, as appropriate. Categorical variables are presented as numbers (percentages), whereas continuous variables are expressed as mean ± standard deviation. To examine the association between NLR status and the rate of cognitive decline, linear mixed-effects models were fitted using the *lmerTest* package in R software, with different cognitive measures as the dependent variables. The main explanatory variables were time (months from baseline), NLR subgroup, and their interaction. The models were adjusted for age, sex, years of education, marital status, and *APOE* ε4 copies, and participant ID was included as a random intercept. For visualization, participants were dichotomized using an NLR of 3.0 to generate Kaplan–Meier survival curves, thereby facilitating an intuitive comparison between the high- and low-inflammatory subgroups. To evaluate progression risk to MCI or AD, two Cox proportional hazards regression models were constructed with NLR status as the primary predictor. The first model was a categorical Cox regression that used dichotomized NLR (≤3.0 versus > 3.0) to estimate supplementary subgroup risk and was adjusted for predefined covariates and baseline global cognition (MMSE). The second model, which served as the primary multivariable Cox regression model, treated NLR as a continuous variable to preserve its full quantitative information while avoiding potential statistical bias associated with arbitrary binary categorization. For all survival analyses, participants without clinical progression were right-censored at their last evaluable visit, and survival time was defined as the interval from baseline to either disease progression or the final follow-up assessment. Only individuals with at least three serial longitudinal evaluations were included in the longitudinal statistical analyses. To investigate whether the association between peripheral inflammation, as measured by the NLR, and cognitive decline was partially explained by core AD pathology, a multiple mediation analysis was performed. In this analysis, baseline NLR served as the independent variable (X), the rate of cognitive decline (estimated from linear regression slopes) served as the dependent variable (Y), and baseline CSF biomarkers (Aβ_42_, t-tau, or p-tau) served as the mediators (M). All continuous variables were standardized to z-scores; therefore, the regression coefficients are presented as standardized β values. The analysis was adjusted for age, sex, education, and *APOE* ε4 carrier status (Model 1). The total, direct, and indirect effects were estimated using Model 4 of the PROCESS macro, implemented through the *bruceR* package in R, with 5,000 bootstrap iterations to obtain robust inference. Subgroup and sensitivity analyses were subsequently conducted using Cox proportional hazards models to evaluate the robustness of the primary findings. Spearman’s rank correlation coefficients were calculated for all pairwise associations and visualized as a correlation heatmap. Two-tailed *p-*values were adjusted for multiple comparisons using the Benjamini–Hochberg false discovery rate (FDR) procedure, which was applied to all unique pairwise correlations. FDR-adjusted *p-*values < 0.05 were considered statistically significant. All statistical analyses were performed using R software (version 4.2.3).

## 3 Results

### 3.1 Baseline participants characteristics

A total of 1,190 participants were included in the study, comprising 275 (23.11%) with high NLR and 915 (76.89%) with normal NLR at baseline. The mean follow-up duration was 4.99 ± 2.99 years, ranging from 6 months to 10 years. The mean baseline age of the cohort was 73.73 ± 7.03 years, and the mean number of *APOE* ɛ4 copies was 0.51 ± 0.65. Most participants were males (56.22%), married (74.71%), White (92.86%), and non-Hispanic (96.64%).

Compared with participants with normal NLR, those with high NLR were significantly older (75.19 ± 6.73 versus 73.29 ± 7.07 years; *p* < 0.001), were more often to be males (64.00% versus 53.88%; *p* = 0.003), and exhibited poorer baseline cognitive performance on RAVLT immediate (35.66 ± 11.74 versus 38.36 ± 11.39; *p* = 0.001), ADNI-MEM (0.41 ± 0.88 versus 0.54 ± 0.84; *p* = 0.024), and ADNI-EF (0.30 ± 0.90 versus 0.46 ± 0.86; *p* = 0.010). Theses participants also exhibited lower CSF Aβ_42_ (1,041 ± 597.9 versus 1,154 ± 620.3; *p* = 0.024) and higher t-tau (288.9 ± 125.7 versus 263.8 ± 116.0; *p* = 0.009) and p-tau (27.91 ± 14.39 versus 25.07 ± 12.93; *p* = 0.008). Furthermore, participants with high NLR demonstrated a significantly higher rate of progression to MCI or AD than those with normal NLR (42.55% versus 30.82%; *p* < 0.001). No significant differences were observed between the two NLR groups with respect to years of education, marital status, *APOE* ɛ4 copies, race and ethnicity.

Within high- and low-NLR groups, cognitively progressive participants carried a significantly greater *APOE* ɛ4 allele burden than cognitively stable participants (both *p* < 0.001). Similarly, all cognitive performance measures were significantly poorer in cognitively progressive subgroups than in the cognitively stable subgroups within both NLR categories (all *p* < 0.001 to *p* = 0.013). Moreover, CSF biomarkers of AD pathology, including Aβ_42_, t-tau, and p-tau, identified significantly greater pathological changes in the cognitively progressive subgroups across both NLR categories (all *p* < 0.001). The clinical and demographic characteristics of the study participants are summarized in Table 1.

**Table 1:** Baseline characteristics of all participants divided groups based on NLR subgroup and diagnostic conversion.

Characteristics	High NLR^1^		Normal NLR^2^		*P* (*U* or *χ*^2^) (*η*^2^), 1 vs. 2	*P* (*U* or *χ*^2^) (*η*^2^), 3 vs. 4	*P* (*U* or *χ*^2^) (*η*^2^), 5 vs. 6
	Cognitively normal^3^	Cognitively progressive^4^	Cognitively normal^5^	Cognitively progressive^6^			
*N*	158	117	633	282	–		
Age (years)	75.11 ± 7.00	75.30 ± 6.37	72.76 ± 7.05	74.46 ± 6.97	<0.001 (3.967) (0.013)	0.822 (0.225) (0.000)	0.001 (3.376) (0.012)
Sex (% male)	103 (65.19)	73 (62.39)	333 (52.61)	160 (56.74)	0.003 (8.799) (0.086)	0.633 (0.228) (0.029)	0.247 (1.340) (0.038)
Education	16.51 ± 2.76	16.15 ± 2.54	16.08 ± 2.87	15.88 ± 2.82	0.053 (1.937) (0.003)	0.269 (1.109) (0.004)	0.485(0.698) (0.001)
Marital status					0.232 (1.430) (0.035)	0.031 (4.637) (0.130)	0.158 (1.992) (0.047)
Married	115	98	459	217	–	–	–
Single	43	19	174	65	–	–	–
*APOE*ɛ4 copies	0.41 ± 0.63	0.78 ± 0.63	0.39 ± 0.58	0.72 ± 0.71	0.104 (1.628) (0.002)	<0.001 (4.765) (0.077)	<0.001 (47264) (0.246)
White (%)	147 (93.04)	113 (96.58)	577 (91.15)	268 (95.04)	0.215 (1.537) (0.036)	0.201 (1.636) (0.077)	0.041 (4.162) (0.067)
Non-Hispanic ethnicity (%)	150 (94.94)	116 (99.15)	611 (96.52)	273 (96.81)	0.926 (0.009) (0.003)	0.052 (3.761) (0.117)	0.826 (0.048) (0.007)
Cognitive performance							
MMSE	28.41 ± 1.58	27.59 ± 1.82	28.38 ± 1.66	27.44 ± 1.86	0.789 (0.268) (0.000)	<0.001 (3.959) (0.054)	<0.001 (62244) (0.303)
ADAS11	7.60 ± 4.38	11.13 ± 4.35	7.52 ± 3.76	11.35 ± 4.84	0.203 (1.273) (0.001)	<0.001 (6.637) (0.139)	<0.001 (47044) (0.472)
ADAS13	12.12 ± 6.48	18.18 ± 6.64	11.85 ± 7.06	18.32 ± 7.06	0.075 (1.781) (0.003)	<0.001 (7.572) (0.174)	<0.001 (42272) (0.522)
ADASQ4	3.98 ± 2.33	6.08 ± 2.57	3.83 ± 2.27	6.12 ± 2.58	0.061 (1.872) (0.003)	<0.001 (7.069) (0.155)	<0.001 (46151) (0.483)
CDR-SB	0.79 ± 0.84	1.48 ± 1.07	0.77 ± 0.84	1.48 ± 1.15	0.158 (1.413) (0.002)	<0.001 (5705) (0.383)	<0.001 (56644) (0.365)
FAQ	1.25 ± 2.49	4.17 ± 4.68	1.17 ± 2.51	4.04 ± 4.70	0.082 (1.743) (0.003)	<0.001 (4998) (0.455)	<0.001 (50626) (0.428)
RAVLT immediate	39.96 ± 12.40	29.86 ± 7.64	40.87 ± 10.95	32.73 ± 10.26	0.001 (3.416) (0.010)	<0.001 (4613) (0.501)	<0.001 (10.57) (0.109)
RAVLT learning	5.10 ± 2.41	3.64 ± 2.46	5.25 ± 2.51	3.55 ± 2.61	0.171 (1.369) (0.002)	<0.001 (4.931) (0.082)	<0.001 (9.377) (0.088)
RAVLT percent of forgetting	43.68 ± 31.95	68.29 ± 31.89	44.07 ± 30.19	66.05 ± 31.93	0.146 (1.455) (0.002)	<0.001 (6.321) (0.128)	<0.001 (9.975) (0.099)
mPACCdigit	−2.83 ± 4.27	−6.62 ± 4.08	−2.88 ± 3.91	−6.66 ± 4.71	0.203 (1.274) (0.001)	<0.001 (7.432) (0.168)	<0.001 (48591) (0.456)
mPACCtrialsB	−2.71 ± 4.00	−6.48 ± 3.95	−2.68 ± 3.73	−6.38 ± 4.52	0.100 (1.648) (0.002)	<0.001 (7.781) (0.182)	<0.001 (47726) (0.465)
LM Delayed Recall	9.71 ± 4.82	5.60 ± 4.49	9.45 ± 4.39	5.76 ± 4.81	0.298 (1.041) (0.001)	<0.001 (7.194) (0.159)	<0.001 (11.37) (0.124)
ADNI-MEM	0.75 ± 0.89	−0.05 ± 0.64	0.76 ± 0.76	0.04 ± 0.79	0.024 (2.258) (0.004)	<0.001 (4155) (0.550)	<0.001 (13.04) (0.157)
ADNI-EF	0.57 ± 0.92	−0.05 ± 0.75	0.61 ± 0.82	0.13 ± 0.85	0.010 (2.574) (0.006)	<0.001 (5486) (0.407)	<0.001 (7.969) (0.065)
ADNI-LAN	0.59 ± 0.90	0.02 ± 0.74	0.57 ± 0.77	0.17 ± 0.80	0.075 (1.785) (0.003)	<0.001 (5688) (0.385)	<0.001 (7.256) (0.055)
ADNI-VS	0.17 ± 0.72	−0.05 ± 0.71	0.10 ± 0.68	−0.08 ± 0.80	0.557 (0.587) (0.001)	0.013 (2.513) (0.023)	0.002 (78328) (0.122)
CSF AD biomarker							
*N*	120	81	465	186	–	–	–
Aβ_42_	1,204 ± 630.5	795.8 ± 447.0	1,278 ± 619.7	842.6 ± 502.7	0.024 (2.263) (0.006)	<0.001 (2650) (0.452)	<0.001 (23020) (0.468)
t-tau	260.3 ± 109.3	331.2 ± 136.8	237.4 ± 99.16	329.6 ± 128.3	0.009 (2.632) (0.008)	<0.001 (3294) (0.322)	<0.001 (22974) (0.468)
p-tau	24.30 ± 11.64	33.26 ± 16.36	22.06 ± 11.04	32.55 ± 14.22	0.008 (2.646) (0.008)	<0.001 (3066) (0.369)	<0.001 (21789) (0.494)

NLR, Neutrophil-to-Lymphocyte Ratio; MMSE, Mini-Mental State Examination; ADAS11, Alzheimer’s Disease Assessment Scale–Cognitive Subscale 11-item version; ADAS13, Alzheimer’s Disease Assessment Scale–Cognitive Subscale 13-item version; ADASQ4, Alzheimer’s Disease Assessment Scale–Delayed Word Recall subscale (Q4); CDR-SB, Clinical Dementia Rating Scale Sum of Boxes; FAQ, Functional Assessment Questionnaire; RAVLT, Rey Auditory Verbal Learning Test; mPACC, modified Preclinical Alzheimer’s Cognitive Composite; LM, Logical Memory; ADNI-MEM, Alzheimer’s Disease Neuroimaging Initiative Memory composite score; ADNI-EF, Alzheimer’s Disease Neuroimaging Initiative Executive Function composite score; ADNI-LAN, Alzheimer’s Disease Neuroimaging Initiative Language composite score; ADNI-VS, Alzheimer’s Disease Neuroimaging Initiative Visuospatial composite score; CSF, Cerebrospinal Fluid; AD, Alzheimer’s Disease; Aβ_42_, Amyloid-beta 42; t-tau, total tau protein; p-tau, phosphorylated tau protein. For a clearer visual representation of pairwise p-value comparisons between groups: 1, High NLR; 2, Normal NLR; 3, Cognitively normal in the High NLR group; 4, Cognitively progressive in the High NLR group; 5, Cognitively normal in the Normal NLR group; 6, Cognitively progressive in the Normal NLR group.

### 3.2 Association between NLR and longitudinal cognitive performance: linear mixed-effects models

Linear mixed-effects models indicated that NLR was markedly associated with accelerated longitudinal cognitive decline. Throughout the follow-up period, participants with elevated NLR experienced a significantly faster rate of global cognitive decline, as measured by the MMSE (*β* = −0.0066; *p* = 0.0025), ADAS-11 (*β* = 0.0159; *p* = 0.0002), and ADAS-13 (*β* = 0.0208; *p* = 0.0001). Elevated NLR was also associated with a more rapid increase in clinical severity, as measured by the CDR-SB (*β* = 0.0033; *p* = 0.0188), and with accelerated decline across multiple cognitive domains, including memory (ADNI-MEM: *β* = −0.0013, *p* = 0.0037; ADAS-Q4: *β* = 0.0031, *p* = 0.0306), executive (FAQ: *β* = 0.0134, *p* = 0.0003; mPACCdigit: *β* = −0.0110, *p* = 0.0027; mPACCtrialsB: *β* = −0.0101, *p* = 0.0031; ADNI-EF: *β* = −0.0014, *p* = 0.0047), language (ADNI-LAN: *β* = −0.0012, *p* = 0.0177), and visuospatial function (ADNI-VS: *β* = −0.0014, *p* = 0.0208; Figure 1 and Table 2).

**Figure 1:**
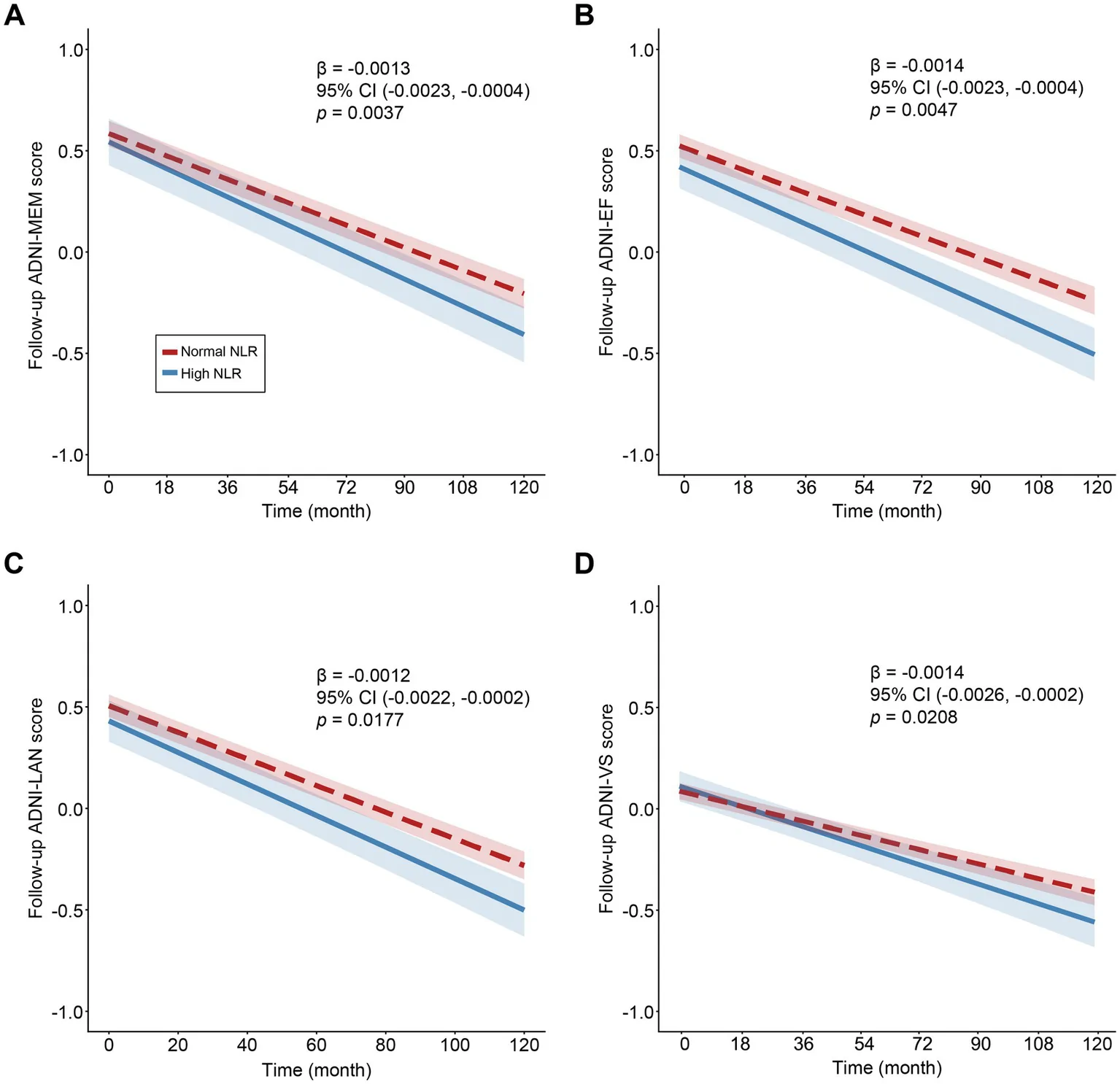
Longitudinal changes in ADNI domain-specific composite scores according to baseline NLR subgroup. Values represent adjusted model predictions derived from linear mixed-effects models, adjusted for age, sex, education, marital status, and *APOE* ε4 copies. Shaded areas denote CIs. **(A)** ADNI-MEM score; **(B)** ADNI-EF score; **(C)** ADNI-LAN score; **(D)** ADNI-VIS score. Among participants with normal NLR levels, the sample sizes were 915, 230, 594, 8, 239, 62, 98 and 94 individuals at the 0-, 18-, 36-, 54-, 72-, 90-, 108- and 120-month follow-up time points. In contrast, the high-NLR subgroup consisted of 275, 74, 165, 3, 73, 16, 26 and 18 subjects at these corresponding visits.

**Table 2:** Summary of linear mixed-effects models.

Predictors	MMSE			ADAS11			ADAS13			ADASQ4		
	Coefficient	SE	*P-*value	Coefficient	SE	*P*-value	Coefficient	SE	*P*-value	Coefficient	SE	*P*-value
Age*time	−0.0010	0.0001	<0.0001	0.0018	0.0003	<0.0001	0.0028	0.0003	<0.0001	0.0009	0.0001	<0.0001
Male*time	0.0063	0.0019	0.0009	−0.0173	0.0037	<0.0001	−0.0248	0.0045	<0.0001	−0.0051	0.0012	<0.0001
Education*time	0.0010	0.0003	0.0034	−0.0011	0.0006	0.0983	−0.0014	0.0008	0.0845	−0.0001	0.0002	0.5291
Married status*time	0.0086	0.0021	0.0001	−0.0133	0.0042	0.0015	−0.0160	0.0051	0.0017	−0.0005	0.0014	0.7446
*APOE* ɛ4 copies*time	−0.0338	0.0015	<0.0001	0.0634	0.0029	<0.0001	0.0799	0.0035	<0.0001	0.0101	0.0009	<0.0001
High NLR*time	−0.0066	0.0022	0.0025	0.0159	0.0043	0.0002	0.0208	0.0052	0.0001	0.0031	0.0014	0.0306

Predictors	CDR-SB			FAQ			RAVLT immediate recall			RAVLT learning across trials		
	Coefficient	SE	*P*-value	Coefficient	SE	*P*-value	Coefficient	SE	*P*-value	Coefficient	SE	*P*-value
Age*time	0.0010	0.0001	<0.0001	0.0026	0.0002	<0.0001	−0.0036	0.0004	<0.0001	−0.0007	0.0001	<0.0001
Male*time	−0.0041	0.0012	0.0008	−0.0072	0.0032	0.0246	0.0149	0.0054	0.0057	0.0037	0.0017	0.0349
Education*time	−0.0008	0.0002	0.0002	−0.0031	0.0006	<0.0001	0.0041	0.0009	<0.0001	0.0001	0.0003	0.8681
Married status*time	−0.0066	0.0014	<0.0001	−0.0179	0.0037	<0.0001	0.0005	0.0061	0.9303	0.0009	0.0020	0.6323
*APOE* ɛ4 copies*time	0.0259	0.0009	<0.0001	0.0607	0.0025	<0.0001	−0.0590	0.0042	<0.0001	−0.0053	0.0013	0.0001
High NLR*time	0.0033	0.0014	0.0188	0.0134	0.0037	0.0003	−0.0081	0.0062	0.1935	−0.0025	0.0020	0.2172

Predictors	RAVLT percent of forgetting			MPACCdigit			MPACCtrialsB			LM Delayed Recall		
	Coefficient	SE	*P*-value	Coefficient	SE	*P*-value	Coefficient	SE	*P*-value	Coefficient	SE	*P*-value
Age*time	0.0088	0.0014	<0.0001	−0.0027	0.0002	<0.0001	−0.0024	0.0002	<0.0001	−0.0024	0.0002	<0.0001
Male*time	0.0232	0.0198	0.2406	0.0134	0.0032	<0.0001	0.0131	0.0030	<0.0001	0.0049	0.0026	0.0648
Education*time	−0.0107	0.0035	0.0020	0.0020	0.0006	0.0003	0.0016	0.0005	0.0016	0.0005	0.0005	0.3192
Married status*time	0.0274	0.0222	0.2164	0.0156	0.0036	<0.0001	0.0136	0.0033	<0.0001	0.0015	0.0030	0.6240
*APOE* ɛ4 copies*time	0.0947	0.0153	<0.0001	−0.0601	0.0024	<0.0001	−0.0558	0.0023	<0.0001	−0.0202	0.0020	<0.0001
High NLR*time	0.0359	0.0229	0.1172	−0.0110	0.0037	0.0027	−0.0101	0.0034	0.0031	−0.0019	0.0030	0.5317

Predictors	ADNI_MEM			ADNI_EF			ADNI_LAN			ADNI_VS		
	Coefficient	SE	*P*-value	Coefficient	SE	*P*-value	Coefficient	SE	*P*-value	Coefficient	SE	*P*-value
Age*time	−0.0004	0.0000	<0.0001	−0.0002	0.0000	<0.0001	−0.0002	0.0000	<0.0001	−0.0001	0.0000	0.0016
Male*time	0.0018	0.0004	<0.0001	0.0017	0.0004	<0.0001	0.0021	0.0004	<0.0001	0.0013	0.0005	0.0190
Education*time	0.0003	0.0001	<0.0001	−0.0001	0.0001	0.4466	0.0000	0.0001	0.8102	0.0000	0.0001	0.8635
Married status*time	0.0008	0.0005	0.0806	0.0022	0.0005	<0.0001	0.0017	0.0005	0.0006	0.0015	0.0006	0.0167
*APOE* ɛ4 copies*time	−0.0068	0.0003	<0.0001	−0.0051	0.0003	<0.0001	−0.0056	0.0003	<0.0001	−0.0039	0.0004	<0.0001
High NLR*time	−0.0013	0.0005	0.0037	−0.0014	0.0005	0.0047	−0.0012	0.0005	0.0177	−0.0014	0.0006	0.0208

All models included the main effects of the following predictors: age, sex, years of education, marital status, *APOE* ε4 allele copies, NLR subgroup, and time since baseline (in months). For brevity, the coefficients for these variables are not presented in the summary table. The reported coefficients are unstandardized and represent the estimated monthly change in the cognitive score per unit change in the predictor.

MMSE Mini-Mental State Examination, ADAS11, Alzheimer’s Disease Assessment Scale–Cognitive Subscale 11-item version; ADAS13, Alzheimer’s Disease Assessment Scale–Cognitive Subscale 13-item version; ADASQ4, Alzheimer’s Disease Assessment Scale–Delayed Word Recall subscale (Q4); CDR-SB, Clinical Dementia Rating Scale Sum of Boxes; FAQ, Functional Assessment Questionnaire; RAVLT, Rey Auditory Verbal Learning Test; mPACC, modified Preclinical Alzheimer’s Cognitive Composite; LM, Logical Memory; ADNI-MEM, Alzheimer’s Disease Neuroimaging Initiative Memory composite score; ADNI-EF, Alzheimer’s Disease Neuroimaging Initiative Executive Function composite score; ADNI-LAN, Alzheimer’s Disease Neuroimaging Initiative Language composite score; ADNI-VS, Alzheimer’s Disease Neuroimaging Initiative Visuospatial composite score; SE, Standard Error.

Consistent with expectations, older age, a greater number of *APOE* ɛ4 copies, and female sex were significantly associated with a faster rate of cognitive decline across all assessments (all *p* < 0.05). In contrast, higher education attainment and married status were significantly associated with a slower rate of cognitive decline, indicating a protective effect (Table 2).

### 3.3 Association between NLR and progression risk: Kaplan–Meier survival analysis and cox proportional hazards models

During the follow-up period, 117 of 275 participants (42.55%) with high NLR progressed to a diagnosis of MCI or AD, compared with 282 of 915 participants (30.82%) with normal NLR. The association between NLR and progression risk to a more impaired diagnostic category was investigated using Kaplan–Meier survival analysis and multivariable Cox proportional hazard models. Kaplan–Meier analysis of dichotomized NLR levels (high versus normal) demonstrated a significantly steeper decline in the cumulative proportion of cognitive stability over time in the high NLR group (*p* < 0.0001; Figure 2). The vertical lines indicate the median time to cognitive progression. Participants with high NLR exhibited a significantly shorter median duration of cognitive stability, reflecting accelerated cognitive decline. In contrast, the cumulative incidence remained below 50% in the normal NLR group throughout the 10-year follow-up period, indicating that lower NLR was associated with a substantially lower long-term risk of progression. The corresponding risk table indicates adequate cohort retention, particularly during the early follow-up period. After adjustment for age, sex, education attainment, marital status, and *APOE* ɛ4 copies, the multivariable Cox proportional hazards model demonstrated that each 1-unit increase in baseline NLR, analyzed as a continuous variable, was associated with a 20% increase in the risk of cognitive progression (hazard ratio [HR] = 1.20, 95% confidence interval [CI]: 1.12–1.29; *p* < 0.001; Figure 3). The proportional hazards assumption was evaluated using Schoenfeld residuals and was satisfied for NLR in the continuous (*p* = 0.568) and dichotomized (*p* = 0.834) models. Although the global test was statistically significant (*p* < 0.001; Supplementary Figure 3), this finding primarily reflected time-varying effects of age and baseline MMSE—which are commonly observed in longitudinal aging cohorts (Guo et al., 2026; Liu and Caselli, 2018). Because the primary exposure variable satisfied the proportional hazards assumption, the Cox model provided valid estimates of the association between NLR and cognitive progression. Collectively, the Kaplan–Meier and Cox regression analyses indicate that elevated NLR is significantly and independently associated with an increased risk of cognitive decline.

**Figure 2:**
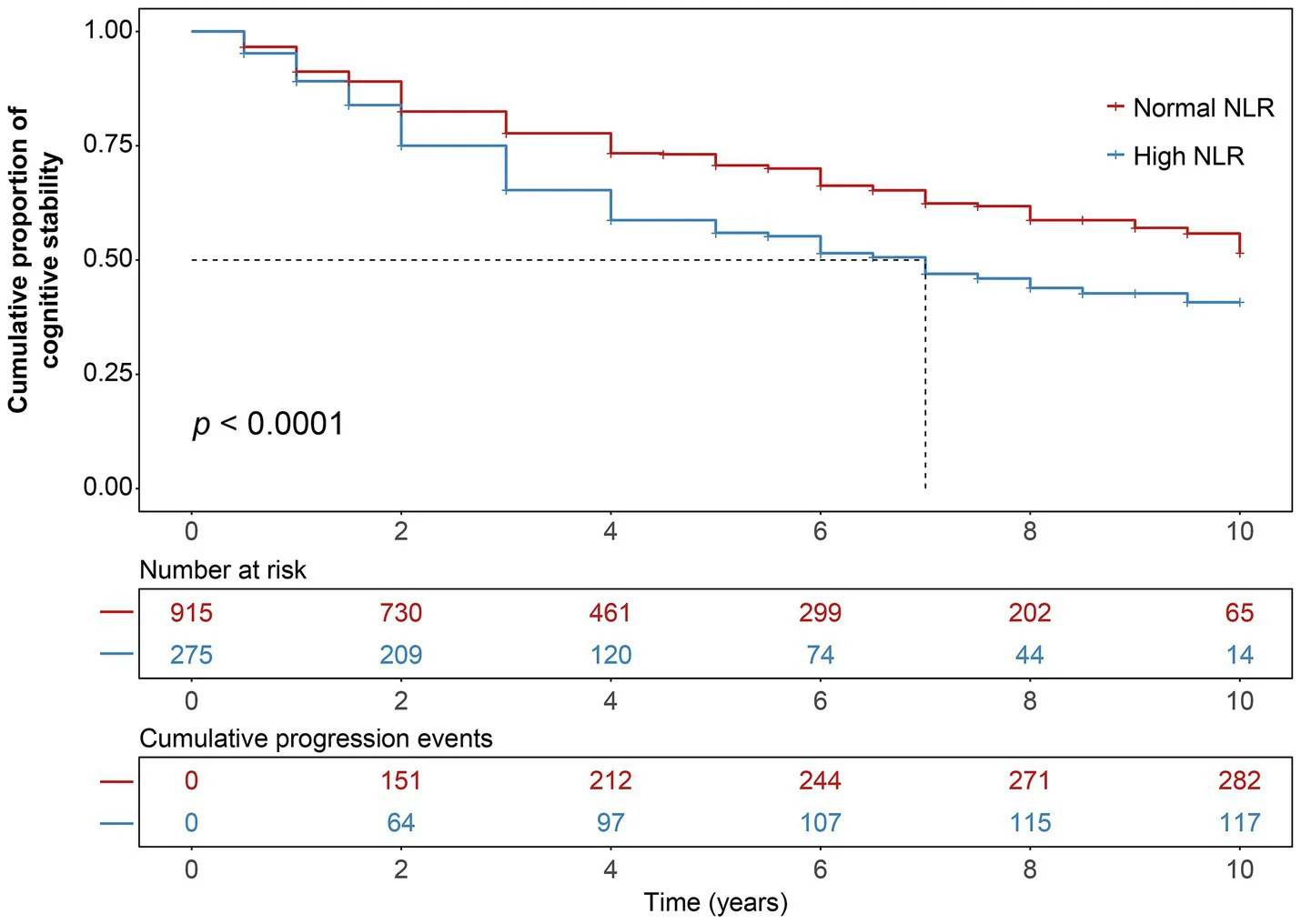
Kaplan–Meier survival curves illustrating time to cognitive progression according to baseline NLR subgroup. The associated table at the bottom presents the number of participants at risk in each subgroup at the specified time points throughout the follow-up period.

**Figure 3:**
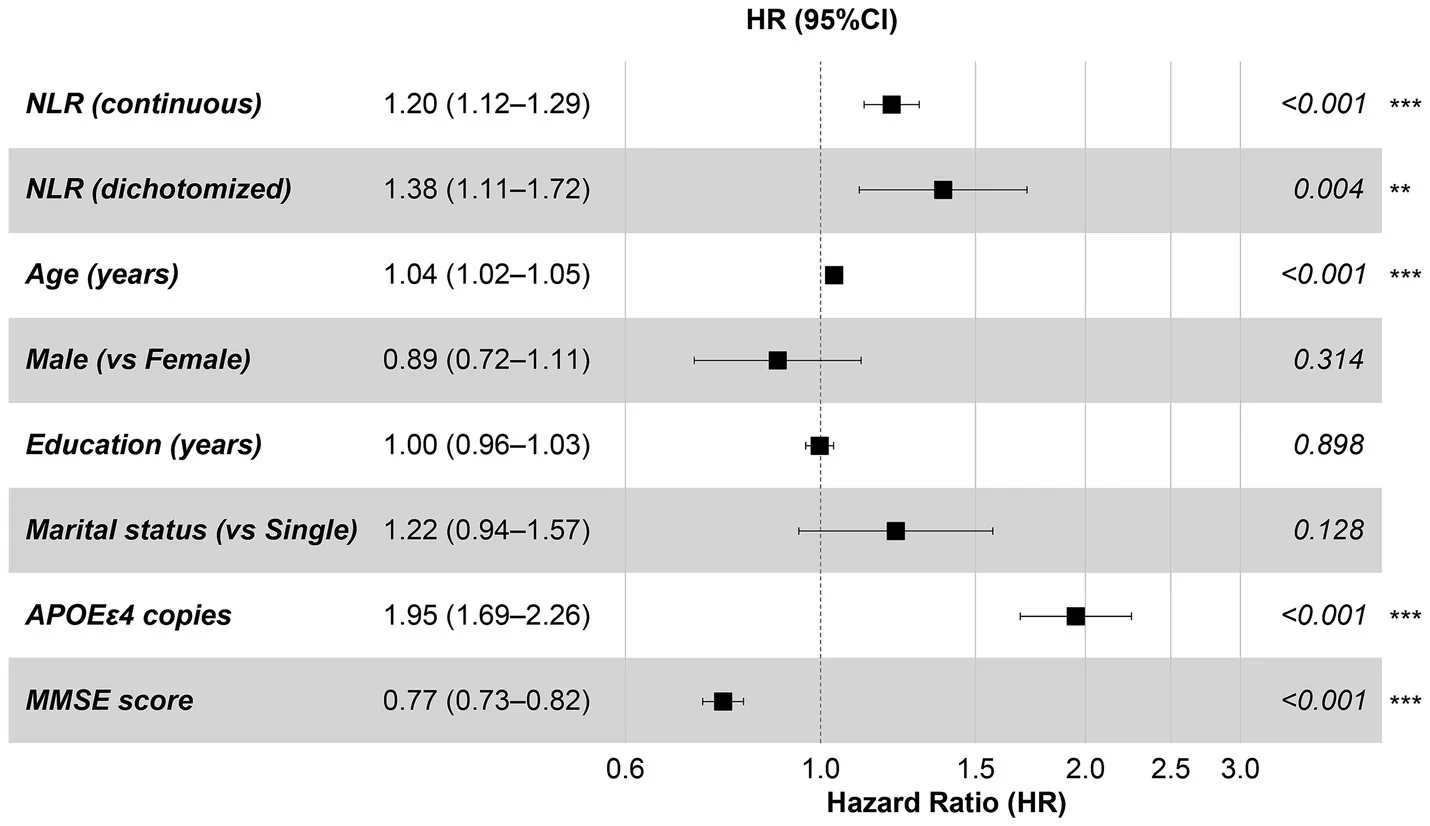
Forest plot of the multivariable Cox proportional hazards model for predictors of cognitive progression. The model included continuous NLR, dichotomized NLR, age, sex, education, marital status, *APOE* ε4 copies, and baseline MMSE. HRs greater than 1 indicate an increased risk of cognitive progression, whereas HR less than 1 indicate a reduced risk of progression. The vertical reference line denotes HR = 1. Statistical significance (*p* < 0.05) is indicated by 95% CIs that do not cross the reference line.

### 3.4 Mediation analyses of AD pathological biomarkers

Mediation analyses indicated that Aβ_42_ was a statistically non-significant contributor of the association between NLR and cognitive decline across multiple cognitive domains. In contrast, t-tau and p-tau each explained a modest proportion of this association (t-tau: 29.6–38.2%; p-tau: 34.5–42.7%; Figure 4). Collectively, these findings indicate that tau pathology accounts for a modest yet significant proportion of the association between NLR and cognitive decline.

**Figure 4:**
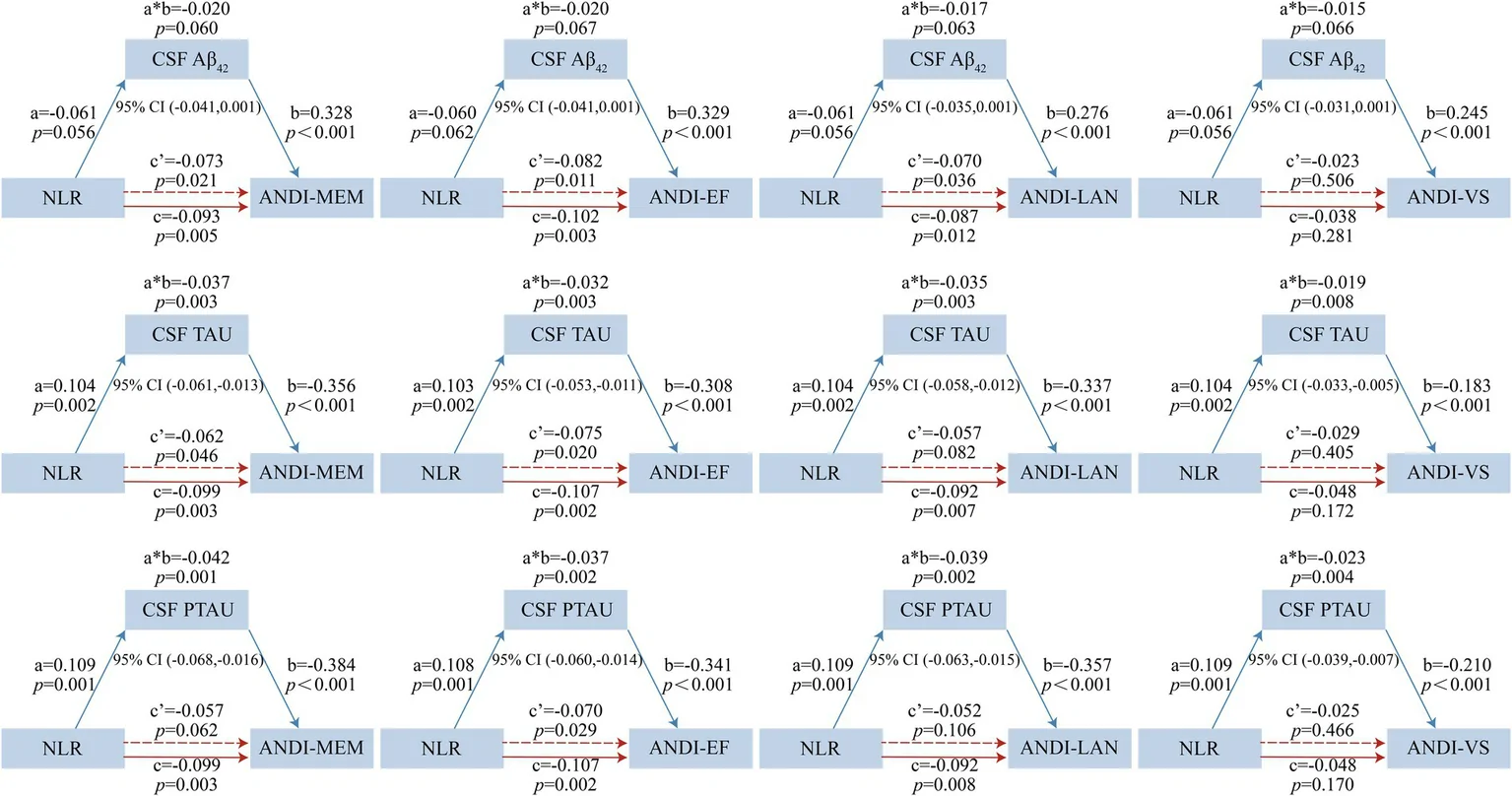
Mediation analysis examining whether AD biomarkers (CSF *Aβ4_2_*, *t-tau*, and *p-tau*) statistically mediate the association between *NLR* and cognitive decline. Standardized path coefficients are presented. Path *a* represents the association between *NLR* and the biomarker; path *b* represents the association between the biomarker and cognitive decline; path *c* represents the total effect of *NLR* on cognitive decline; path *c′* represents the direct effect. The indirect effect (*a* × *b*) is presented with bias-corrected bootstrapped 95% *CIs* based on 5,000 resamples. All models were adjusted for age, sex, education, marital status, and *APOE ε4* copies.

### 3.5 Subgroup and sensitivity analyses

The association between NLR and cognitive progression risk remained consistent with the primary analysis across all subgroups stratified by age, sex, education, marital status, *APOE* ε4 carrier status, and baseline cognitive status, with statistically non-significant interaction effects observed (all *p-*values for interaction > 0.05; Supplementary Figure 1). Sensitivity analyses further evaluate the robustness of the primary findings. These analyses sequentially excluded early progressors (participants who progressed within the first 0.5 years), removed the analysis to participants with extreme NLR values (above the 99th percentile), restricted the analysis to participants with at least 1 year of follow-up, and applied a simplified adjustment model including only age, sex, education, and *APOE* ε4 copies. In addition, alternative NLR cutoffs of 2.5 and 3.5 were evaluated alongside the primary cutoff of 3.0. Across all sensitivity analyses, the association between elevated NLR and an increased risk of cognitive progression remained materially unchanged (Supplementary Figure 2).

### 3.6 Correlations of NLR with demographic, neuropsychological, and CSF biomarkers of AD

A correlation heatmap was constructed to examine the cross-sectional associations of baseline NLR with demographic, neuropsychological, and CSF biomarkers of AD (Figure 5). NLR was significantly and positively correlated with age, male sex, *APOE* ɛ4 copies, multiple cognitive assessment measures reflecting cognitive decline, including ADAS11, ADAS13, ADAS-Q4, CDR-SB, FAQ, and RAVLT Percent of Forgetting, as well as CSF t-tau and p-tau (all *p* < 0.05). Positive correlations are represented by a red color gradient, with correlation coefficients ranging from 0 to 1. In contrast, NLR was significantly and negatively correlated with marital status, RAVLT Immediate, RAVLT Learning, mPACCdigit, mPACCtrialsB, LM Delayed Recall, ADNI-MEM, ADNI-EF, ADNI-LAN and CSF Aβ_42_ (all *p* < 0.05). Negative correlations are indicated by a blue color gradient, with correlation coefficients ranging from −1 to 0. Education level, MMSE, and ADNI-VS exhibited non-significant correlations with NLR, as indicated by correlation coefficients close to zero and the absence of a notable color gradient in the correlation matrix (Figure 5). Given the cross-sectional design, these correlations should be interpreted as baseline phenotypic alignments rather than evidence of underlying biological mechanisms.

**Figure 5:**
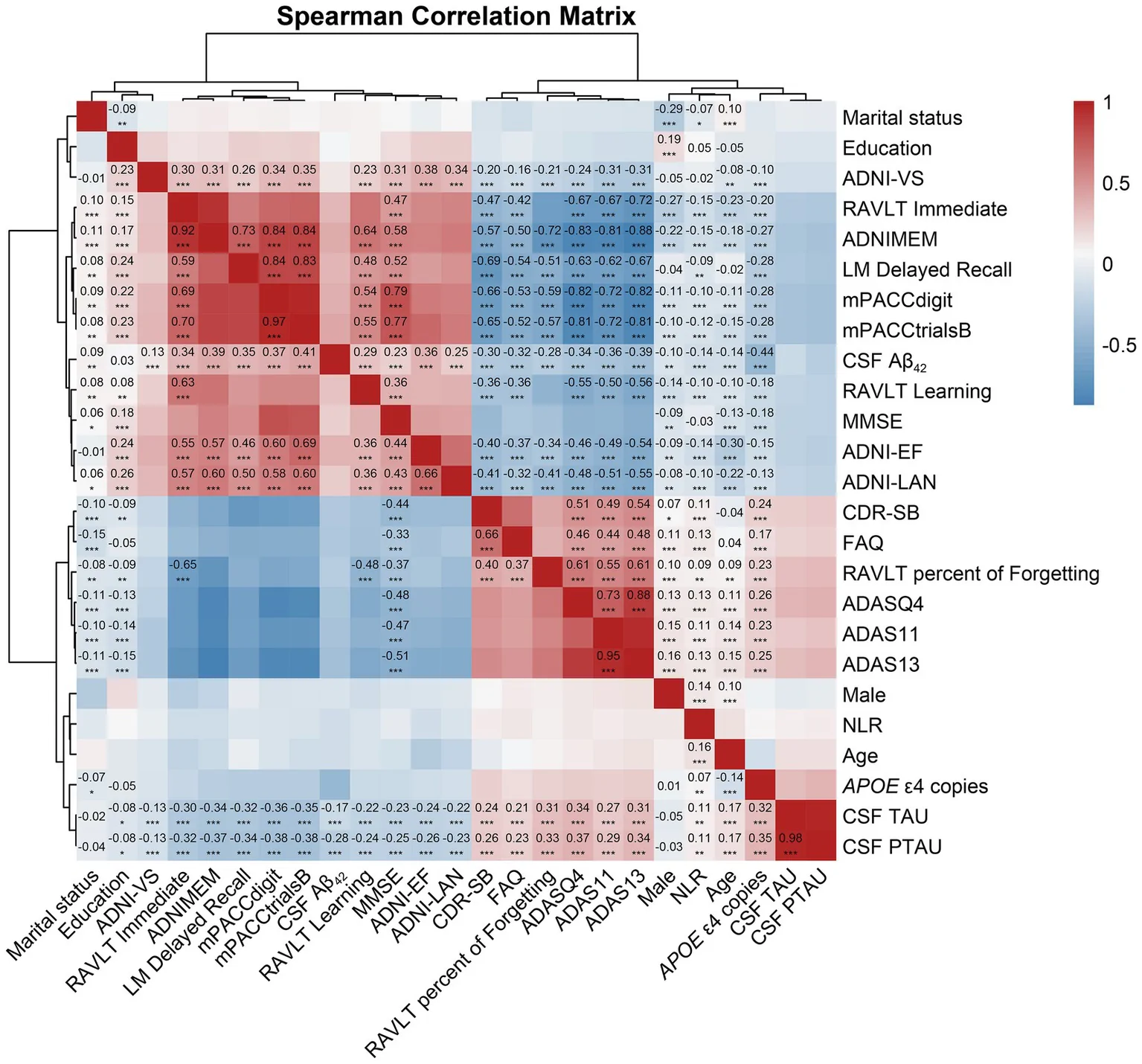
Spearman correlation matrix illustrating baseline NLR with demographic characteristics, cognitive measures, and CSF biomarkers. Ellipses represent the direction and magnitude of the correlations. With red indicating positive correlations and blue indicating negative correlations. Correlation strength increases as the ellipses become more elongated. Statistical significance was determined after adjustment of two-tailed *p*-values using the Benjamini–Hochberg FDR procedure. Asterisks indicate FDR-adjusted significance levels (*p < 0.05, **p < 0.01, ***p < 0.001).

## 4 Discussion

This study comprehensively investigated the association of baseline NLR with longitudinal cognitive decline and progression risk to MCI or AD in a well-characterized cohort of non-people living with dementia older adults from the ADNI database. Elevated baseline NLR was independently associated with accelerated deterioration across multiple cognitive domains, including global cognition, memory, executive function, language, and visuospatial function, after adjustment for potential confounding factors, including age, sex, education level, and *APOE* ε4 carrier status. Furthermore, a high NLR level emerged as a significant independent risk factor for cognitive progression, with each 1-unit increase in NLR associated with a 20% higher risk of progression to MCI or AD. Mediation analyses further indicated that CSF tau pathology (t-tau and p-tau), but not Aβ_42_, statistically accounted for a modest proportion (29.6–42.7%) of the association between NLR and cognitive decline. These observations are consistent with the hypothesis that peripheral immune dysregulation and tau pathology are interrelated during the early stages of AD; however, they do not establish a causal pathway. Moreover, subgroup and sensitivity analyses confirmed the robustness of the primary findings, demonstrating that the association between NLR and cognitive impairment remained stable across demographic and clinical subgroups. Collectively, these findings suggest that elevated NLR is a clinically accessible peripheral marker associated with early cognitive decline and tau pathology in older adults at risk of AD. Although NLR may help identify individuals at increased risk, these observational data neither establish NLR as a validated predictive biomarker nor support a causal contribution to disease progression.

Neuroinflammation is recognized as a key driver of neurodegenerative diseases and has been extensively investigated in AD (Heneka et al., 2024). Owing to the presence of the BBB, the CNS is considered an immune-privileged site that, under physiological conditions, effectively restricts the entry of most peripheral immune cells. Nevertheless, persistent inflammatory conditions can compromise BBB integrity, leading to increased permeability, immune cells infiltration into the CNS, and exacerbation of neuroinflammation, thereby directly contributing to neuronal damage and synaptic alterations (Daneman and Prat, 2015). NLR is an easily accessible and cost-effective prognostic marker of systemic inflammatory status. By integrating information from neutrophils and lymphocytes, NLR may better reflect the systemic immune burden than either parameter alone (Wang et al., 2025). Several cross-sectional studies have reported elevated NLR levels in patients with AD compared with healthy controls (Evlice et al., 2023; Algul and Kaplan, 2024; Giannelli et al., 2023). Nonetheless, most of these studies focused on individuals with established AD, in whom age-related comorbidities and advanced neuropathology may have confounded the findings. In contrast, the present study included a non-people living with dementia cohort comprising CN and early MCI participants, enabling evaluation of the association between NLR and subsequent cognitive decline before overt dementia onset. Although one prospective study suggested that elevated NLR may increase the risk of cognitive impairment, its limited sample size warrants further validation of this association (Cervellati et al., 2024). The present study comprehensively assessed multiple cognitive domains using a battery of neuropsychological tests and demonstrated that elevated NLR was not only associated with global cognitive decline but also with impairments in specific domains, including memory and executive function. Furthermore, mediation analyses evaluated the extent to which core AD pathological biomarkers statistically accounted for the association between NLR and cognitive decline. Collectively, these findings provide additional insights into the relationship between peripheral immune dysregulation and central neurodegenerative processes during the early stages of AD. However, this study’s results do not constitute a validated predictive framework.

The potential biological mechanisms linking elevated NLR to accelerated cognitive decline primarily involve peripheral immune imbalance, systemic inflammation, and neuroinflammation-mediated AD pathology. As a marker reflecting the balance between innate and adaptive immunity, an elevated NLR indicates a shift toward a pro-inflammatory state characterized by increased neutrophil counts and relative lymphopenia (Buonacera et al., 2022). Under pathological conditions associated with BBB disruption, neutrophils, the principal effector cells of innate immunity, can infiltrate the brain parenchyma, including the hippocampus and cortex. Following entry into the brain, neutrophils release pro-inflammatory mediators, including myeloperoxidase, neutrophil gelatinase-associated lipocalin, and reactive oxygen species (ROS), thereby triggering and amplifying neuroinflammation, which contributes to neuronal dysfunction and cognitive impairment (Baik et al., 2014; Bawa et al., 2020; Aries and Hensley-McBain, 2023; Wu et al., 2020). Consistent with these mechanisms, AD mouse models exhibited increased formation of neutrophil extracellular traps, which contribute to BBB disruption and neuronal damage (Manda-Handzlik and Demkow, 2019). Furthermore, neutrophils from patients with AD exhibit enhanced adhesion to cerebral capillary endothelia, further compromising BBB integrity and exacerbating neuroinflammation (Aries and Hensley-McBain, 2023). Neutrophil accumulation has also been associated with capillary stalls, resulting in reduced local cerebral perfusion and aggravated cognitive deficits in animal models (Cruz Hernández et al., 2019). Conversely, neutrophil depletion reduces AD-like neuropathology and improves memory performance in mice (Zenaro et al., 2015). Moreover, neutrophils may further promote AD pathogenesis by releasing specific proteins, such as CAP37, that activate microglia (Kong et al., 2020).

Meanwhile, reduced peripheral lymphocytes reflect impaired adaptive immune function. Several mechanisms may contribute to peripheral lymphocyte depletion in AD. First, BBB disruption in patients with AD facilitates the entry of peripheral immune cells, particularly CD8^+^ T cells, into the CNS, thereby exacerbating neuroinflammation and accelerating disease progression (van Olst et al., 2022). Second, elevated neutrophil levels promote excessive ROS production, resulting in lymphocyte DNA damage and cell death (Hou et al., 2022). Third, peripheral lymphocytes from patients with AD exhibit increased susceptibility to apoptosis (Eckert et al., 2001). The mediation findings are consistent with the possibility that peripheral immune imbalance, as reflected by elevated NLR, coincides with tau pathology and is statistically associated with cognitive decline. Tau-related pathology may represent one of several pathways through which peripheral immune dysregulation and central neurodegenerative processes are cross-sectionally linked during the early stages of AD. Previous studies indicate that activated microglia promote tau phosphorylation and the propagation of neuroinflammation by releasing pro-inflammatory cytokines and chemokines (Maphis et al., 2015; Lee et al., 2015). In turn, abnormal tau aggregation can activate microglia, establishing a positive feedback loop that further exacerbates neuroinflammation and neurodegeneration (Didonna, 2020). Nevertheless, these observational data cannot determine whether elevated NLR precedes or contributes to tau accumulation or whether both markers are attributed to shared underlying disease mechanisms. Reverse causality, including the possibility that early tau pathology influences peripheral immune function, and residual confounding, remain plausible alternative explanations. Accordingly, a hypothetical framework is proposed in which systemic immune imbalance, reflected by higher NLR, coincides with increased tau pathology, and both markers are jointly associated with subsequent cognitive decline in AD.

It is important to interpret these statistical associations within their clinical context. Although the estimated regression coefficients for NLR in linear mixed-effects models were modest in absolute magnitude, their cumulative impact over time may be clinically meaningful in chronic neurodegenerative diseases. Specifically, each 1-unit increase in baseline NLR was associated with an additional annual decline of approximately 0.07 points on the MMSE and 0.21 points on the ADAS-13. Over a typical 5-year follow-up period, these estimates correspond to between-group differences of approximately 0.35 MMSE points and 1.05 ADAS-13 points, respectively. Although these differences may appear modest at the individual level, they are comparable to effect sizes reported for established AD risk factors, including *APOE* ε4 carriage, in population-based cohorts (Qian et al., 2021; Zhang et al., 2020). Moreover, in the Cox proportional hazards model, each 1-unit increase in NLR was associated with a 20% higher hazard of progression to MCI or AD (HR = 1.20, 95% CI: 1.12–1.29). Given that NLR is inexpensive, widely available, and potentially modifiable through lifestyle or pharmacological interventions, even modest population-level effect sizes may yield substantial public health benefits.

This study has several notable strengths that enhance the reliability and potential clinical applicability of its findings. Using a large-scale, multicenter, and longitudinal ADNI cohort, with up to 10 years of follow-up and repeated cognitive assessments, enabled a robust evaluation of cognitive trajectories and the predictive value of the NLR while overcoming key limitations inherent to cross-sectional designs. Furthermore, the analyses incorporated comprehensively adjustment for demographic, lifestyle, and genetic factors, including *APOE* ε4 carrier status, and the robustness of the observed associations was confirmed by extensive sensitivity and subgroup analyses. Notably, the inclusion of CN participants and participants with MCI enabled evaluation of the potential predictive value of NLR during the preclinical and early symptomatic stages of AD, a period that is particularly relevant for early risk identification. However, this study has some limitations that warrant careful consideration. First, the cohort consisted predominantly of non-Hispanic White older adults, which may limit the generalizability of the findings to other racial and ethnic groups. Second, the mediation analyses are subject to important methodological limitations. The proposed pathway, in which peripheral inflammation, as reflected by NLR, is associated with tau pathology and subsequent cognitive decline, should be regarded as a hypothetical framework rather than an empirically established mechanism. Because NLR, CSF biomarkers, and baseline cognitive function were assessed at approximately the same time point, temporal sequencing cannot be established, thereby precluding causal inference. Alternative explanations, including reverse causality, whereby early tau pathology alters peripheral immune profiles, and shared underlying disease severity, remain equally plausible. Third, although the models were adjusted for core demographic and clinical covariates, including age, sex, education, marital status, *APOE* ε4 carrier status, and baseline MMSE, several systemic conditions known to influence NLR could not be considered. Specifically, NLR is a nonspecific inflammatory marker that can be elevated by acute infections, obesity, diabetes mellitus, cardiovascular disease, smoking, corticosteroid therapy, and other chronic inflammatory disorders. Detailed information on these comorbidities—particularly medication use, including statins, immunosuppressants, or anti-inflammatory agents; smoking history; and subclinical metabolic disturbances—were unavailable in the ADNI dataset. Consequently, residual confounding from these unmeasured or incompletely captured factors cannot be excluded and may have contributed to the observed associations. Accordingly, the mediation findings should be interpreted as hypothesis-generating and require validation in future studies with comprehensive comorbidity and medication data, serial biomarker measurements, and, ideally, interventional designs.

In conclusion, this study identifies NLR as a practical and cost-effective marker associated with early cognitive progression in AD. The observed statistical association between NLR and tau pathology is consistent with the potential relevance of peripheral immune dysregulation to early disease trajectories, thereby providing a rationale for future studies to develop and validate formal predictive models incorporating NLR alongside other established risk factors.

## Data Availability

The datasets presented in this study can be found in online repositories. The names of the repository/repositories and accession number(s) can be found at: data for the current study were obtained from the Alzheimer’s Disease Neuroimaging Initiative (ADNI) database (adni.loni.usc.edu).

## Supplementary material

SUPPLEMENTARY FIGURE 1 Subgroup analysis of the association between baseline NLR and cognitive progression. The diamond represents the overall adjusted HR, whereas squares represent subgroup-specific HRs. Subgroups were defined according to age (<75 versus ≥75 years), sex (female versus male), education (≤median versus >median), marital status (married versus single), *APOE*4 status (non-carrier versus carrier), and baseline diagnosis (CN versus MCI). The *p*-value for interaction assesses the heterogeneity of the association between NLR and cognitive progression across subgroups.

SUPPLEMENTARY FIGURE 2 Sensitivity analyses of the association between baseline NLR and cognitive progression under different analytical scenarios: (1) Primary analysis with continuous NLR; (2) exclusion of early progressors (≤0.5 years); (3) exclusion of participants with follow-up < 1 year; (4) exclusion of extreme NLR values (> 99th percentile); (5) NLR dichotomized at 3.0; and (6) a simplified adjustment model including only age, sex, education, and *APOE* ε4 copies. All analyses, except scenario (6), were adjusted for baseline MMSE score, age, sex, education, *APOE* ε4 copies, and marital status. Black squares represent the point estimate (HRs), and the horizontal black lines indicate the corresponding 95% CIs.

SUPPLEMENTARY FIGURE 3 Schoenfeld residual plots for NLR in continuous and dichotomized Cox proportional hazards models. **(A)** Smoothed Schoenfeld residuals for continuous NLR over time. **(B)** Smoothed Schoenfeld residuals for dichotomized NLR over time. In both panels, the solid line represents the fitted trend of the residuals, and the dashed lines represent the corresponding confidence bands. The horizontal dotted line at zero indicates no time-dependent effect. The absence of systematic trends away from zero supports satisfaction of the proportional hazard assumption for NLR in both model specifications (continuous: *p* = 0.568; dichotomized: *p* = 0.834).

## Glossary

Aβ: Amyloid-beta
Aβ_42_: Amyloid-beta 42
AD: Alzheimer’s disease
ADAS11: Alzheimer’s Disease Assessment Scale-Cognitive Subscale 11-item version
ADAS13: Alzheimer’s Disease Assessment Scale-Cognitive Subscale 13-item version
ADAS-Cog: Alzheimer’s Disease Assessment Scale-Cognitive Subscale
ADAS-Q4: Alzheimer’s Disease Assessment Scale Delayed Word Recall subscale
ADNI: Alzheimer’s Disease Neuroimaging Initiative
ADNI-EF: ADNI composite score for executive function
ADNI-LAN: ADNI composite score for language
ADNI-MEM: ADNI composite score for memory
ADNI-VIS: ADNI composite score for visuospatial function
*APOE*: Apolipoprotein E
BBB: Blood–brain barrier
CBC: Complete blood count
CDR: Clinical Dementia Rating
CDR-SB: Clinical Dementia Rating Scale Sum of Boxes
CI: Confidence interval
CNS: Central nervous system
CN: Cognitively normal
CSF: Cerebrospinal fluid
DNA: Deoxyribonucleic acid
FAQ: Functional Assessment Questionnaire
HR: Hazard ratio
MCI: Mild cognitive impairment
MMSE: Mini-Mental State Examination
mPACCdigit: Modified Preclinical Alzheimer’s Cognitive Composite with Digit Symbol Substitution
mPACCtrialsB: Modified Preclinical Alzheimer’s Cognitive Composite with Trails B
NLR: Neutrophil-to-lymphocyte ratio
PACC: Preclinical Alzheimer’s Cognitive Composite
p-tau: Phosphorylated tau
RAVLT: Rey Auditory Verbal Learning Test
SD: Standard deviation
t-tau: total tau
